# Effect of Degree of Milling (DOM) on Physicochemical and Nutritional Quality of Selected Rice Variety (BRRI dhan78)

**DOI:** 10.1155/ijfo/6034633

**Published:** 2025-06-27

**Authors:** Sharmin Akter, Rokeya Begum, Sharifa Sultana Dipti, Md. Abdul Alim, Somaiya Islam Shuchy, Habibul Bari Shozib, Md. Rakibul Hasan

**Affiliations:** ^1^Department of Food Technology and Nutritional Science, Mawlana Bhashani Science and Technology University, Tangail, Bangladesh; ^2^Grain Quality and Nutrition Division, Bangladesh Rice Research Institute (BRRI), Gazipur, Bangladesh; ^3^Department of Nutrition and Food Engineering, Daffodil International University, Dhaka, Bangladesh

**Keywords:** blood glucose response, BRRI Dhan78, degree of milling, glycemic index, nutrients

## Abstract

Rice, a staple for over half the global population, undergoes milling that removes bran and germ, impacting its nutrient content, physical properties, and glycemic response. The study was conducted to estimate the effects of the degree of milling (DOM) on the physicochemical, nutritional quality, blood glucose response (GR) and glycemic index (GI) of the BRRI dhan78. Rice samples collected from BRRI were milled at 0% (brown rice), 5% (partial milled), and 10% (full milled). Brown rice had the highest ash (1.3%), crude fiber (1.4%), protein (9.4%), and fat content (1.3%), while full milled rice had the highest moisture (11.1%) and carbohydrate content (79.7%). Thiamin content was affected by DOM in a linear fashion, declining from 0.15 mg/100 g to 0.13 mg/100 g as DOM increased. Significant reductions in Fe and Zn were observed as the DOM increased, from 8.6 mg/kg to 3.7 mg/kg and 22.0 mg/kg to 14.2 mg/kg, respectively. The length and breadth ratio increased along with the increase in DOM from 2.3 to 2.8. The *L*∗ and *h* values of different milled rice increased significantly (*p* < 0.05) from 61.8 to 72.7 and 79.1 to 88.9, respectively, with increasing DOM, while *a*∗, *b*∗, and *c*∗ values significantly (*p* < 0.05) decreased from 4.5 to 0.3, 23.4 to 14.0, and 23.8 to 13.6, respectively. Springiness and cohesiveness and of cooked rice significantly (*p* < 0.05) increased from 0.2 to 0.5 and from 0.2 to 0.3, while hardness and chewiness decreased from 3.3 × 10^4^ to 2.2 × 10^4^ N/m^2^ and from 8.1 × 10^2^ to 2.7 × 10^2^ N/m^2^, respectively, with an increase in DOM. Full milled rice caused the highest glucose increase at different time intervals while brown rice caused the lowest. Brown rice had the lowest GI (54.4), while full milled rice had the highest GI (75.9). Brown rice was slightly liked, while partial milled rice was moderately liked. Both offer healthier benefits than full milled rice based on the DOM.

## 1. Introduction

Rice (*Oryza sativa* L.), a staple food that is extremely important to over half of the world's population, is grown in more than 100 countries as a staple food, and more than 96% of the land was used for “cereal agriculture” in Bangladesh alone [1]. With an average per-capita consumption rate of 144.5 kg/year, Bangladesh is the third-largest rice producer in the world as of 2022–2023 and is essential to the Bangladeshi diet, contributing 75% of daily calories and 55% of protein [2]. Among the numerous rice varieties, BRRI dhan78, developed by the Bangladesh Rice Research Institute (BRRI), is recognized for its nutritional quality, yield (4.5–7 tonnes per hectare), adaptability to saline environments, and resistance to pests and diseases [3]. This high-yielding, salt-tolerant variety is primarily characterized by its medium-grain size and high amylose content, which influences its cooking and eating quality [4]. Prior to consumption, rice undergoes a certain amount of milling. The amount of bran powder removed from dehusked rice is measured and referred to as the degree of milling (DOM). The DOM affects the quality of rice as it determines how much of the rice bran and germ layers are removed during processing [5]. These outer layers are rich in essential nutrients like dietary fiber, vitamins (especially B vitamins like thiamin), minerals (such as iron and zinc), and healthy fats [6]. As the DOM increases (more bran removal), the nutrient-rich layers are stripped away, leading to a significant reduction in the nutritional content of the rice [7]. Thus, higher DOM increases the amount of carbohydrates while lowering the amounts of fats, proteins, fiber, vitamins, and minerals [8–11]. Consumer acceptance of the organoleptic quality is anticipated when milling to a 6%–8% degree of polishing, which preserves roughly 60% iron and zinc [12]. Thus, rice could be processed with less bran layer removal to obtain the greatest nutritional value. Brown rice, with minimal milling (0% milling), retains most of its bran and germ, offering higher fiber and nutrient content. However, it also has a harder texture and longer cooking time, making it less popular among consumers [13]. Furthermore, milled rice exhibits a higher glycemic index (GI) due to the removal of the bran layer, which slows down starch digestion ([14]. The prevalence of diabetes varies greatly in Bangladesh; a systematic review published data ranging from 4.5% to 35.0% [15]. According to Cho et al. [16], 13.7 million people in Bangladesh could have diabetes by 2045. One factor fueling this epidemic is high carbohydrate consumption, which makes up 70%–80% of total calories in many Southeast Asian countries [17]. The relationship between DOM and rice's GI is particularly important, as a higher glycemic response is associated with an increased risk of Type 2 diabetes and cardiovascular diseases [18]. Thus, understanding how different milling degrees affect the nutritional profile and health outcomes is crucial for consumers seeking healthier dietary options. The current study is aimed at investigating the effects of different DOMs on the nutrient composition and different quality parameters in BRRI dhan78 as well as the effects of milling on the blood glucose response (GR) and GI of this variety.

## 2. Materials and Methods

### 2.1. Study Design

**Figure 1:**
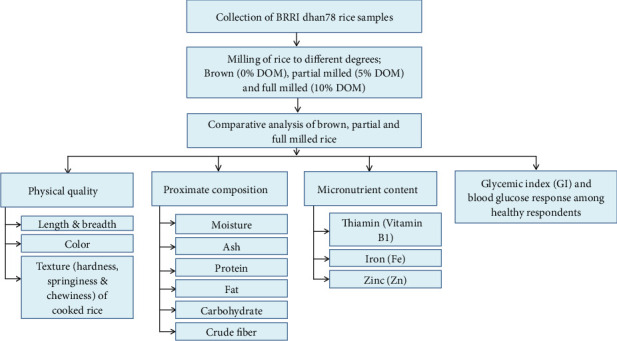
Study design.

The study design in Figure 1 focused on the comparative analysis of BRRI dhan78 rice samples, milled to different degrees: brown rice (0%), partial milled rice (5% of DOM), and full milled rice (10% of DOM). The rice samples undergo multiple analyses to assess the changes due to different DOMs across four main areas: physical quality, proximate composition, micronutrient content, and GI along with blood GR.

### 2.2. Collection, Milling, and Storage of Rice Samples

Samples of BRRI dhan78 rice were collected from the BRRI, Gazipur, Dhaka. The DOMs of rice were determined according to the method by Dipti et al. [12]. Outer husks were removed from dried paddy using a Satake Testing Husker (Model THU-35B, Satake Corporation, Hiroshima, Japan) with rubber rollers coated with polyvinyl chloride compound to avoid mineral contamination. The dehusked brown rice was milled using a Grainman tester mill (Model 60-220 50-DT, Grain Machinery Manufacturing Corporation, Miami, Florida, United States). Three different DOMs were tested: 0%, 5% and 10%, which were mentioned as brown rice, partial milled, and full milled rice, respectively. The DOM was calculated by the following equation:

(1) $$\begin{array}{c} \text{Degree of milling }\left(\%\right)=\frac{\text{weight of milled rice }\left(\text{g}\right)}{\text{weight of brown rice }\left(\text{g}\right)}\times 100. \end{array}$$

After milling, the samples were sealed in ziplock bags and stored at room temperature in a desiccator until further analysis. All analytical chemicals and standards used in this research were obtained from Merck (Merck KGaA, Darmstadt, Germany).

### 2.3. Analysis of Physical Quality

#### 2.3.1. Length and Breadth (LB) Measurement

LB was measured in millimeter using a slide caliper. Before and after every measurement was taken with the slide caliper, the instrument was “zeroed” and locked to ensure precise measurements. The LB was determined by using the following formula [13]:

(2) $$\begin{array}{c} \frac{L}{B}=\frac{\text{Length}}{\text{Breadth}}. \end{array}$$

#### 2.3.2. Texture Analysis

A texture analyzer (Imada, Japan) was stacked with 5 g of trigger force that was used to measure the features of texture of different degrees of milled cooked rice, as described by Wang et al. [19]. The probe of the texture analyzer was an aluminum cylindrical probe (P/36R). A sample of 0 mm in height was loaded on the desk and then subjected to two cycles of compression up to 20% of the original height using a 20-mm cylindrical probe at a speed of 1.0 mm/s, a trigger force of 5 g, and a recovery period between compressions of 15 s. The parameters of hardness, springiness, and chewiness were determined.

#### 2.3.3. Color Measurement

The color quality of the samples was evaluated by a chroma meter (CR-400; Konica Minolta, Tokyo, Japan). The color was measured in terms of hunter lab coordinates, namely, lightness (*L*∗), redness (*a*∗), yellowness (*b*∗), chroma (*c*∗), and hue (*h*). The instrument was calibrated with a standard white tile (*Y* = 85.1, *X* = 0.3171, *y* = 0.3234) before measurements. Nearly 50 g of rice was held within a container at a constant depth of 5 cm. The sensor probe connected to the chroma meter was gently placed into the sample. The total color difference (Δ*E*) between samples and the whiteness value (*W*) was calculated using the following formulas [20]:

(3) $$\begin{array}{c} \Delta E=\sqrt{{\left(\Delta L*\right)}^{2}+{\left(\Delta a*\right)}^{2}+{\left(\Delta b*\right)}^{2}}, \end{array}$$

(4) $$\begin{array}{c} W=\sqrt{{\left(100-L*\right)}^{2}+a{*}^{2}+b{*}^{2}}. \end{array}$$

### 2.4. Analysis of Proximate Parameter

#### 2.4.1. Moisture

The moisture content of different degrees of milled rice samples was determined according to AOAC method [21]. A crucible was weighed, and about 3 g of the sample was added. The sample was dried at 105°C for 4 h, cooled in a desiccator, and reweighed. Drying continued in 30-min intervals until the weight stabilized, and the moisture content was calculated using following formula:

(5) $$\begin{array}{c} \text{Moisture}\left(\%\right)=\frac{\text{Sample weight before drying}-\text{Sample weight after drying}}{\text{Sample Weight before drying}}\times 100. \end{array}$$

#### 2.4.2. Ash

Ash content was determined following AOAC method [21]. About 3 g of the dried sample was placed in a preweighed crucible and heated at 600°C for 6 h in a muffle furnace (JSMF-45 T). The crucible was then cooled in a desiccator and reweighed to calculate the ash content using the following formula.

(6) $$\begin{array}{c} \text{Ash }\left(\%\right)=\frac{\text{Amount of the ash}}{\text{Sample Weight }}\times 100. \end{array}$$

#### 2.4.3. Fat

Fat content was determined using AOAC method [21]. About 5 g of the sample was wrapped in filter paper, placed in a Soxhlet extractor, and extracted with 250 mL of petroleum ether. After heating for 1–2 h, the solvent was evaporated, and the extracted oil was dried at 80°C–90°C. The beaker was cooled in a desiccator and reweighed to calculate the fat content using the following formula:

(7) $$\begin{array}{c} \text{Fat }\left(\%\right)=\frac{\text{Weight of beaker with fat}-\text{Weight of beaker}}{\text{Sample Weight }}\times 100. \end{array}$$

#### 2.4.4. Crude Fiber

Crude fiber content was determined using AOAC method [21]. About 5 g of moisture and fat-free sample was boiled in 1.25% sulfuric acid for 30 min and then filtered and washed. The residue was boiled in 1.25% NaOH for 30 min, filtered, and washed again. The residue was dried at 105°C, cooled, and weighed (*A*). It was then ashed at 600°C, cooled, and reweighed (*B*). Fiber content was calculated using following formula:

(8) $$\begin{array}{c} \text{Fiber }\left(\%\right)=\frac{A-B}{\text{Weight of moisture and fat}-\text{free sample taken }}\times 100. \end{array}$$

#### 2.4.5. Protein

Protein content was determined with the Kjeldahl method [1, 22]. About 0.54 g of the sample was digested with sulfuric acid and a copper catalyst to convert nitrogen to ammonium sulfate. After digestion, the solution was made alkaline and ammonia was distilled into 0.1 N H_2_SO_4_. The ammonia was titrated with 0.1 N NaOH using methyl red as an indicator. Nitrogen content was calculated, and protein content was obtained by multiplying nitrogen by a factor of 5.95.

#### 2.4.6. Carbohydrate

The carbohydrate content of rice samples was calculated from the percentage of other components of that sample by subtracting the additive value of moisture, ash, fat, protein and fiber from 100 [1].

(9) $$\begin{array}{c} \text{Carbohydrate }\left(\%\right)=100-\left[\text{Moisture }\left(\%\right)+\text{Ash }\left(\%\right)+\text{Fat }\left(\%\right)+\text{Protein }\left(\%\right)+\text{Fiber}\left(\%\right).\right. \end{array}$$

### 2.5. Analysis of Micronutrients

#### 2.5.1. Fe and Zn

Fe (milligrams per kilogram) and Zn (milligrams per kilogram) were measured using an Inductively Coupled Plasma Optical Emission Spectrometer (ICP-OES) (Shimadzu Model 9820), and rice samples were digested using a Milestone microwave digester at the Rice Analytical Laboratory (RAL) of BRRI, Gazipur, according to the method of AOAC [22]. One gram of dried rice powder sample was taken from a plastic ziplock package and weighed using an analytical balance. The rice sample was placed into a microwave vessel with mixed 6 mL of HNO_3_ and 1 mL of H_2_O_2_. Microwave digestion program was carried out with parameters shown in Table 1. The digested contents were transferred into polypropylene vials and diluted to a fixed volume with deionized water to ensure complete digestion of the rice sample following the microwave digestion program.

Shimadzu ICPE-9820 was coupled with a mini plasma torch, a concentric nebulizer, and a cyclonic chamber. The detailed instrument configuration and operational parameters for analysis are summarized in Table 2. Calibration curves for Fe and Zn were established for quantitative analysis (Figures S2 and S3, respectively).

Concentrated nitric acid (65%) and hydrogen peroxide (30%) used were in trace metal grade. Ultrapure water was from the Milli-Q water-purification system. External calibration standards were prepared by mixing single elemental stocks from Merck and Sigma-Aldrich. Calibration standards were prepared in 1% nitric acid (Table 3). Mix periodic table mix for ICP (Supelco) was used for standard solution.

#### 2.5.2. Thiamin

The analysis of water-soluble B vitamin (Thiamin) content in the rice samples was carried out using an ultrapressure liquid chromatography (UPLC) at RAL of BRRI, Gazipur. This water-soluble vitamin was extracted from the rice powder by acid hydrolysis followed by enzymatic hydrolysis. Briefly, 2 g of the powder of the rice samples was weighed into a 250-mL Erlenmeyer flask (in triplicates). Acid hydrolysis was carried out by adding 40 mL of 0.1 M HCl and heating the mixture at 90°C for 30 min. Thereafter, enzymatic hydrolysis was conducted by adding taka-diastase and heating at 50°C for 2 h. After cooling, the samples were taken in the tubes and centrifuged at 4000 rpm for 5 min. The supernatant was filtered through a 0.45-*μ*m PVDF syringe filter and collected into HPLC auto-sampler vials. The aqueous extract is injected onto a reverse phase C_18_ HPLC column. The fluorescence of thiamin is determined after post column derivatization with alkaline potassium ferricyanide that converts the thiamin to thiochrome. The method is described for the determination of thiamin and riboflavin in rice by HPLC in ASEAN Manual of Food Analysis [23]. The calibration curve for vitamin B1 (thiamin) is presented in Figure S1.

**Figure 2:**
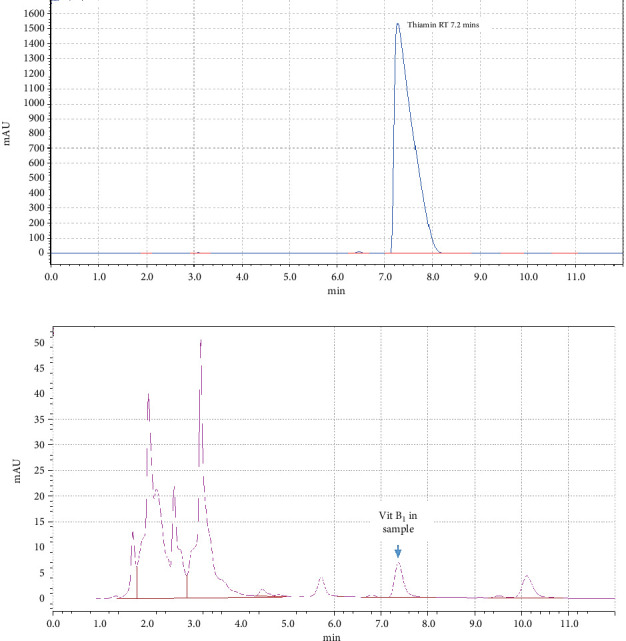
Chromatographs of (a) thiamin standard and (b) thiamin in rice sample.

Thiamin hydrochloride analytical standard (Sigma-Aldrich) was used for standard solution. Extracts from all samples were prepared in triplicate. The calibration standard concentrations for thiamine analysis were prepared at four levels (Table 4) to establish a reliable calibration curve. The fluorescence intensity was measured at an excitation wavelength of 360 nm and emission wavelength of 435 nm.  Figure 2a,b presents the chromatographs of thiamin standard and thiamin extracted from sample, respectively.

### 2.6. Determination of GI

#### 2.6.1. Study Design and Ethical Considerations

An in vivo experiment was conducted at student's residential Hall in Mawlana Bhashani Science and Technology University, Santosh, Tangail. This study was carried out following approval from the Ethical Review Committee of Mawlana Bhashani Science and Technology University (Ethical Approval Number—2023/ERC/007).

#### 2.6.2. Preparation of Test Food

The test foods included brown rice (0% milling), partially milled rice (5%), and fully milled rice (10%) of the BRRI Dhan 78 variety, with pure glucose as the reference food (RF). Raw rice portions were calculated to provide 50 g of available carbohydrates per serving, accounting for differences in carbohydrate content and accounting for minor starch losses during washing (typically < 1%–2% of total starch). The minimum required raw weights were 67.15 g for brown rice, 67.40 g for 5% milled rice, and 64.31 g for 10% milled rice. Each sample was weighed, washed twice with water, boiled until soft, and served at room temperature.

#### 2.6.3. Determination of Blood GR

The blood glucose was determined in 10 apparently healthy volunteers aged 23–27 years given a serving of the sample containing 50 g of available CHO after an overnight fast (10–12 h) [24, 25]. The inclusion criteria for the study involved selecting apparently healthy volunteers aged between 23 and 27 years, with no known chronic conditions such as diabetes, cardiovascular diseases, or metabolic disorders. Participants were required to fast for 10–12 h prior to the test, maintaining a normal body mass index (BMI) within the range of 18.5–24.9 kg/m^2^ (Table S1). Only individuals who willingly consented to participate and follow the test protocol were included in the study. The exclusion criteria comprised individuals with a history of chronic illnesses, including diabetes and cardiovascular disease. Additionally, individuals unable to fast for the required 10–12 h due to health or personal reasons were also excluded from the study. Subjects were requested to consume the test rice with 250 mL of plain water (for the protocol of the test rice) or the glucose in 250 mL of water (for the protocol of the RF) in random order at a comfortable place within 10 min. Blood samples were taken at baseline (Time 0) and at specified intervals for 2 h. For GI determination, the test and RFs were given to the volunteers, and glucose was fed to each volunteer at least two times during the study period, respectively. Finger-prick blood samples were taken from each study participant at baseline and every 30 min during the study period. Finger-prick blood samples were obtained with autolet lancets. Blood samples were analyzed for glucose by the CERA-CHEK 1070 blood glucose monitoring system. The incremental area under the curve (IAUC) for each food, ignoring the area below the fasting level, was calculated geometrically [25]. The mean IAUC in glucose (millimoles per liter) after the test food was expressed as a percent of the mean IAUC after the RF (Table S3). The glycemic load (GL) was also calculated for each rice sample (Table S2).

(10) $$\begin{array}{c} GI=\frac{\text{Incremental area under }2\text{ h plasma glucose curve for test food }}{\text{Incremental area under }2\text{ h plasma glucose curve for }50\text{ g glucose }}\times 100. \end{array}$$

### 2.7. Determination of Sensory Acceptability

#### 2.7.1. Sample Preparation for Sensory Analyses

Samples of different degrees of milled rice were preweighed into 600 g portions and stored in double plastic bags (ziploc) at room temperature until sample preparation and evaluation. The 600-g portions of rice were rinsed three times with cold water that covered the rice, strained to remove excess water, and transferred to a preweighed rice cooker insert bowl. Water was added to a 1:1.3 ratio (w/w) of rice to water, accounting for the water retained from washing rice, and cooked to completion, as indicated by the automatic shift of the cooker to the warm setting, which held an additional 10–15 min at the setting. The top 1 cm layer of cooked rice and rice adhering to the sides of the cooker was not used for texture evaluation. Cooked rice for sampling was taken from the middle of the pot, transferred to a prewarmed (120°C) glass bowl, and gently mixed to avoid kernel breakage. A test portion (≈50 g) of cooked rice was transferred to each of 10 individual prewarmed (120°C) 170 g capacity glass custard cups (Anchor Hocking).

Ten semitrained panelists, comprising the descriptive sensory analysis panel, participated in the tests. Each panelist was previously trained in the principles and concepts of sensory profile analysis. The sensory profile included a seven point hedonic scale (Table 5).

### 2.8. Statistical Analysis

Data analysis was performed using the Statistical Package for the Social Sciences (SPSS, Version 23.0, SPSS Inc., Chicago, Illinois, United States). A one-way analysis of variance (ANOVA) was used to calculate the significant difference (5%) between the rice samples. Graphical representations were created with Microsoft Excel Version 10.0. Both the 95% confidence interval and the significance level were set at 0.05. Each experiment was conducted in triplicate, and the results are presented as mean ± SD.

## 3. Results and Discussion

### 3.1. Physical Properties

Table 6 presents the impact of the DOM on the physical qualities of different levels of milled rice from the BRRI dhan78 variety. The L/B of brown, partial, and full milled rice were 2.3, 2.5 and 2.8, respectively. Table illustrates the significant positive correlation between rising DOM and the L/B value, with a range of 2.3 to 2.8. This may be because the width of the kernels decreased due to the shearing action as the milling process progressed [13].

The overall color change of the kernels was significantly influenced by the different DOMs. Result revealed that, in contrast to *a*∗, *b*∗ and *c*∗ value, which decreased, *L*∗ and *h* value of milled rice increased significantly with increasing of DOM. The color difference (Δ*E*) values of 9.3 for partial milled rice (5%of DOM) and 15.0 for full milled rice (10% of DOM) compared to brown rice suggest notable perceptual changes due to the milling process. Milled rice exhibited higher *L*∗ values (69.6 and 72.7) than brown rice (61.8) which indicates an increase in lightness, this can be associated with the removal of the bran and husk layers during milling, enhancing the visual appeal of the rice. Furthermore, the whiteness values (*W*) also increased from 55.0 for brown rice to 64.1 and 69.3 for milled rice (5% and 10% of DOM, respectively), indicating improved esthetic quality. Table 2 also presents the textural profile of cooked rice where the hardness of the rice decreases significantly with an increase in the DOM, with brown rice exhibiting the highest hardness (3.3 × 10^4^ N/m^2^) compared to milled rice (5% and 10% of DOM), which recorded values of 3.1 × 10^4^ N/m^2^ and 2.2 × 10^4^ N/m^2^, respectively. This reduction in hardness might be attributed to the removal of bran and germ layers during milling, which contain fibrous components that contribute to the overall structural integrity of the rice kernel [26]. Similarly, the chewiness of the cooked rice significantly increased with higher milling degrees, indicating a more palatable texture as milling progresses. However, the springiness values remained consistent at 0.2 for brown and partial milled rice (5% DOM), while it increased to 0.5 for full milled rice (10% DOM), suggesting improved elasticity of the rice texture after extensive milling. Furthermore, cohesiveness increased slightly from 0.2 for brown and partial milled rice to 0.3 for full milled rice, indicating that the more highly milled rice has a better ability to hold together during chewing.

### 3.2. Proximate Composition

Table 7 illustrates the effects of the DOM on the proximate composition of BRRI dhan78 rice variety, with significant differences observed across different milling levels (*p* < 0.05). Proximate composition was analyzed to understand the basic differences among rice samples with various DOM (Table 7). The moisture content of the milled rice (5% DOM) and milled rice (10% DOM) was not significantly different but the moisture content of brown rice was (10.28%) significantly lower from others. Rosniyana et al. [27] reported 10.5% moisture in brown rice, and 10.9% in milled rice (6% DOM) of the Q34 variety, which is the nearest value of 5% degree of milled rice of the selected rice variety. The moisture content of the three studied samples was in the range of 10.3% to 11.1% which is safe for storage.

The handling of samples in the lab and some of the moisture content in the ziplock bags during packaging could be the cause of the variations in moisture content among the rice varieties. The protein content of all the samples is significantly different from each other. The highest crude protein percentage was found in brown rice (9.4%) and the lowest in 10% degrees milled rice (8.0%) of the selected rice variety. A previous study [27] demonstrated that the protein content of the Q3 variety was not significantly different from each other with increasing DOM, but the selected rice variety lost a significant amount of protein content with increasing DOM. As the DOM increases, more of the nutrient-rich bran and germ are removed during the milling process, leading to a reduction in protein content, as these parts of the grain are higher in protein compared to the starchy endosperm [28].

The crude fat content of all the samples is significantly different. The highest fat percentage was found in brown rice (1.3%) and the lowest in 10% degrees milled rice (0.2%). These outcomes are consistent with past research by Oko and Ugwu [29], who obtained fat content from five different rice varieties ranging from 0.5% to 3.5%. The present study demonstrated that brown rice had the highest amount of fiber (1.4%) and 10% degrees of milled rice had the least amount (0.3%). The almost identical crude fiber content of brown rice was reported by Wang et al. [19] to be 1.6%, partially milled rice to be 0.8%, and fully milled rice to be 0.7%. The bran and germ contain a greater proportion of fat, so their removal results in decreased fat content in the milled rice [26]. Additionally, crude fiber, predominantly found in the bran, also diminishes with higher DOM, as the outer layers providing this essential nutrient are stripped away. The ash content of all the samples is significantly different from each other. The highest ash percentage was found in brown rice (1.3%) and the lowest in 10% degree of milled rice (0.7%). As illustrated in Table 3, there is a significant variation in the carbohydrate content among all the samples. The percentage of carbohydrates ranged from 76.3% to 79.7%. Eighty percent of the rice endosperm is made up of carbohydrates, primarily starch. Mostly found in the endosperm is starch. Since milling does not destroy the endosperm or remove other vital elements from the bran layer, the amount of carbohydrates increases as the degree of DOM increases [26].

### 3.3. Micronutrient Content

**Figure 3:**
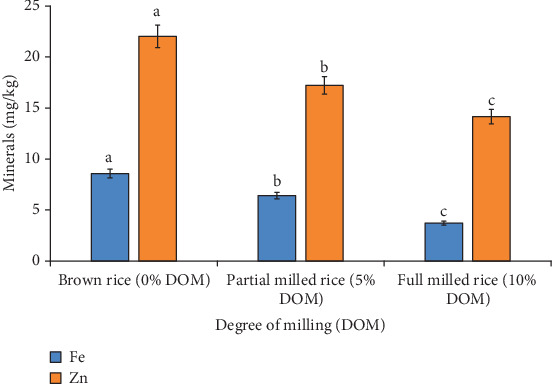
Effect of DOM on Fe and Zn of different degrees of milled rice of BRRI dhan78 variety; values are mean ± standard deviation of three observations; different alphabets presented on each bar indicate significant differences at *p* < 0.05.

Figure 3 illustrates the mineral contents (Fe and Zn) of three different levels of milled rice. The Figure showed that mineral content of rice is significantly affected by DOM. Brown rice (0% of DOM) had the most minerals and full milled rice (10% of DOM) had the least (each of Fe and Zn). As DOM increases, the iron content of the rice sample dramatically drops from 8.6 to 3.7 mg/kg and the zinc content drops from 22.0 to 14.2 mg/kg.

According to Rosniyana et al. [27], the closest value of 5% degree of milled rice of a chosen rice variety was 6.1 mg/kg iron in brown rice and 2.9 mg/kg in partially milled rice and 1.6 mg/kg in fully milled rice. Compared to partially milled and fully milled rice, brown rice had a higher mineral content. According to other research, the majority of minerals displayed a distribution resembling that of total ash [30]. It should be noted that the degree of polishing has a significant effect on the quality and nutritional aspects of white rice grain, thus affecting the essential minerals.

**Figure 4:**
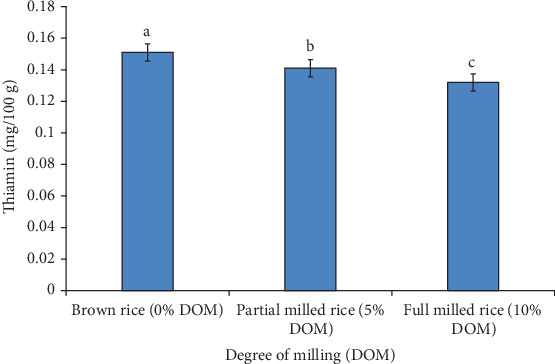
Effect of DOM on thiamine content of different degrees of milled rice of BRRI dhan78 variety; Values are mean ± standard deviation of three observations; Different alphabets presented on each bar indicate significant differences at *p* < 0.05.

Figure 4 presents the thiamine content of three different milled rice samples. The thiamin content of studied samples decreased with the increase of DOM (Figure 4). The highest concentration of thiamin (0.15 mg/100 g) was found in brown rice, while the lowest concentration was found in full milled rice (0.13 mg/100 g). A significant variation was observed in the thiamine content among all the samples.

Previous study also revealed similar results and the method was unable to detect the contents of thiamin when the DOM was above 8% and 6% [19]. It is possible that the uneven distribution of various vitamins in the various layers of bran caused the variation in vitamin loss following each increase in DOM. The testa and aleurone of Simiao rice had higher concentrations of thiamin. This implies that DOM should be avoided if the rice's thiamin content is to be maintained. The observed changes in micronutrients (thiamin, Fe, and Zn, across different DOMs, can be attributed to the removal of the bran and germ layers, which are rich sources of these nutrients. As the DOM increases, the milling process strips away these nutrient-dense outer layers, resulting in a notable decrease in thiamin content. Similarly, iron and zinc levels decreased significantly. The reduction in these micronutrients suggests that higher milling levels lead to lower nutritional quality, potentially impacting the overall health benefits of rice as a dietary staple.

### 3.4. GI and Blood GR Analysis

The results in Table 8 demonstrate that the DOM significantly impacts the glycemic response of the BRRI dhan78 rice variety. Brown rice (0% of DOM) exhibited the lowest glycemic area (148.5 mmol/L·min) and GI (54.4), categorizing it as a low-GI food. In contrast, as the DOM increased, both the glycemic area and GI also increased, partial milled rice showed a glycemic area of 184.5 mmol/L·min and a GI of 67.6, and full milled rice exhibited the highest glycemic area (207.0 mmol/L·min) and GI (75.9). This suggests that the removal of the bran layer during milling results in a higher rate of glucose absorption, making the rice more rapidly digestible and leading to higher glycemic responses.

**Figure 5:**
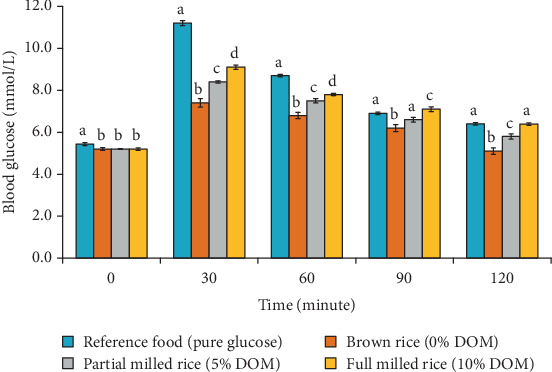
Effect of DOM on blood glucose response of brown, partial milled and full milled rice with reference food (pure glucose); values are mean ± standard deviation of three observations; different superscript letter in different bars at different time intervals indicates significant differences at *p* < 0.05.

Frequent consumption of high-GI foods like fully milled rice can lead to impaired glucose regulation, increasing the risk of developing Type 2 diabetes due to elevated blood sugar spikes over time.  Figure 5 presents the effect of different DOMs on the blood GR of brown rice (0% DOM), partial milled (5% DOM), and full milled (10% DOM) rice compared with a RF (pure glucose) over a 120-min period. At 0 min, all rice samples exhibited similar initial glucose levels, with the RF showing a slightly higher value (5.4 mmol/L) compared to the rice samples, which were all around 5.2 mmol/L.

As time progressed, the blood GR varied significantly among the different rice types. At 30 min, the RF had the highest glucose level (11.2 mmol/L), indicating a rapid rise in blood glucose due to its high GI. Among the rice samples, the brown rice exhibited the lowest increase (7.4 mmol/L), followed by partial milled rice (5% DOM) at 8.4 mmol/L and full milled rice (10% DOM) at 9.1 mmol/L. This suggests that increased DOM correlates with a higher glycemic response, with 10% degree of milled rice causing a more pronounced increase in blood glucose. At 60 min, the RF continued to show the highest blood glucose level (8.7 mmol/L), while brown rice remained the lowest among the rice samples (6.8 mmol/L). The 5% and 10% degree of milled rice samples demonstrated higher values at 7.5 mmol/L and 7.8 mmol/L, respectively, further indicating that reduced milling (i.e., brown rice) helps moderate blood glucose levels over time. By 90 and 120 min, the blood glucose levels had generally declined for all samples. However, brown rice consistently showed the lowest levels, while full milled rice remained the highest. At 120 min, both brown rice and partial milled rice showed similar values (5.1 and 5.8 mmol/L, respectively), while full milled rice still exhibited a higher glucose level of 6.4 mmol/L, comparable to the RF.

### 3.5. Sensory Quality of Cooked Rice

Table 9 presents the sensory evaluation of cooked rice samples which were milled at different degrees for BRRI dhan78 variety, focusing on key attributes such as appearance, taste, texture, chewiness, and overall acceptability. The data indicate that partially milled rice (5% DOM) consistently received the highest ratings across most sensory attributes. Specifically, it scored the highest in appearance (7.5), texture (6.9), and overall acceptability (7.5).

Fully milled rice (10% DOM) followed closely, with slightly lower scores in appearance (7.2) and overall acceptability (7.3), although it was rated highest for taste (6.9) and matched the chewiness score of 5% DOM rice (7.1). Brown rice (0% DOM) was rated the lowest across all attributes, with appearance and texture scoring 6.4, and taste receiving the lowest score (5.4). Despite its lower scores, brown rice still achieved a reasonable level of acceptability (6.5), suggesting that while milled rice varieties may be preferred for their enhanced sensory qualities, brown rice remains an acceptable option, especially considering its additional health benefits. The results underscore the trade-off between sensory appeal and nutritional value, with partially milled rice offering a balanced compromise.

### 3.6. Correlation Analysis

**Figure 6:**
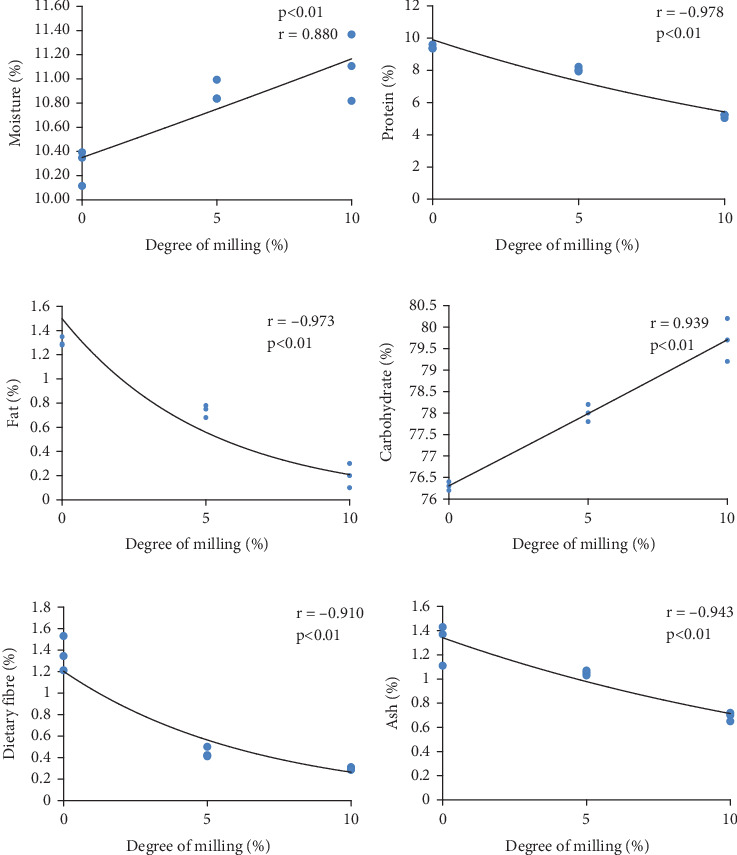
(a–f) Correlation between degree of milling and proximate composition.

**Figure 7:**
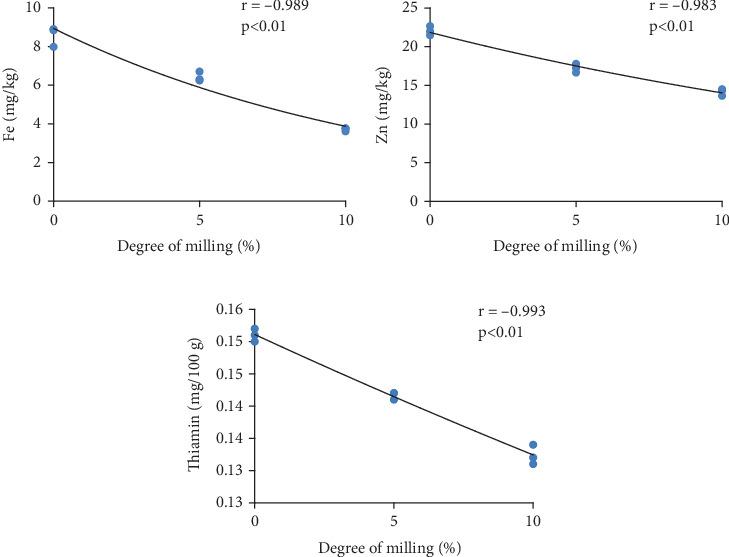
(a–c) Correlation between degree of milling and micronutrients.

Figures 6 and 7 show the correlations between the DOM and various proximate compositions and micronutrient levels in BRRI dhan78 rice, respectively. The results indicate a strong and significant impact of milling on both proximate composition and micronutrient content.

For moisture content, there is a positive correlation, meaning that as the DOM increases, the moisture content also rises. This may occur because milled rice tends to absorb more water due to the removal of outer layers, which may increase its porosity and water-binding capacity [31]. In contrast, protein, fat, crude fiber, and ash show strong negative correlations with DOM. This indicates that the more the rice is milled, the more these nutrients are lost. Milling removes the bran and germ, which are rich in proteins, fats, crude fiber, and ash. This reduction in nutrient content is expected since these components are concentrated in the outer layers of the rice grain, which are removed during the milling process. Carbohydrate content, on the other hand, shows a strong positive correlation with DOM. As the DOM increases, the carbohydrate content increases, likely because the remaining endosperm is predominantly starch. The removal of other nutrient-dense layers results in a relative increase in the carbohydrate content per unit weight.

Micronutrients, for example, Fe, zinc, and thiamin all exhibit strong negative correlations with DOM. This indicates that increasing milling significantly reduces the levels of these vital micronutrients. These nutrients are concentrated in the bran and germ, which are progressively removed as milling increases [32]. Thiamin, in particular, is highly sensitive to milling because it is predominantly found in the aleurone layer and germ, which are stripped away during the process, leading to a sharp decline in its concentration [33].

## 4. Conclusion

Nutrient loss during rice processing is a global public health concern, particularly regarding phenolic compounds and essential nutrients. The DOM plays a critical role in both the GI and nutritional composition of rice. This study revealed that higher DOM leads to greater losses of key nutrients such as proteins, fat, fiber, vitamins, and minerals, particularly thiamin (B1), iron (Fe), and zinc (Zn). Furthermore, increased DOM resulted in higher GI with lighter-colored rice and altered texture profiles, with lower hardness and chewiness but higher springiness and cohesiveness. From a practical perspective, consuming low-milled rice offers significant health benefits due to its higher nutrient retention. The findings suggest that increasing awareness around the consumption of minimally milled rice could improve public health by reducing the risk of noncommunicable diseases associated with high-GI foods. In addition, this research provides valuable intuitions for rice millers, as minimizing DOM could lower processing costs, reduce the percentage of broken kernels, and facilitate the development of functional rice products. Future research should focus on strategies to retain nutrients during milling and explore innovative processing methods to enhance the nutritional value of rice. Promoting the use of low-milled rice and improving milling technology could contribute to sustainable food practices and better health.

## Figures and Tables

**Table 1:** Microwave digestion program.

**Step**	**Time (min)**	**Power**	**Temperature (°C)**
1	15	1800 W	Ramp to 200
2	15	1800 W	Hold at 200
3	5	Cool down	

**Table 2:** Instrument configuration and ICP parameters.

**Parameter**	**Settings**
Torch	Mini torch
Nebulizer	Concentric type
Chamber	Cyclonic type
Peristaltic pump	Inside
Radio frequency power (kW)	1.20
Plasma gas (L/min)	10.0
Auxiliary gas (L/min)	0.60
Carrier gas (L/min)	0.60
View direction	Axial/radial
Exposure time (s)	15

**Table 3:** Calibration standard concentration (micrograms per kilogram).

**Elements**	**Standard 1**	**Standard 2**	**Standard 3**	**Standard 4**	**Standard 5**	**R** ^2^	**Wavelength (nm)**
Fe	80	400	800	2000	4000	0.999	238.2
Zn	80	400	800	2000	4000	0.999	213.8

**Table 4:** Calibration standard concentration (micrograms per milliliter).

**Vitamin**	**Standard 1**	**Standard 2**	**Standard 3**	**Standard 4**	**R** ^**2**^	**Fluorescence excitation wavelength**	**Fluorescence emission wavelength**
Thiamin	0.0625	0.125	0.25	0.5	0.9993	360 nm	435 nm

**Table 5:** Hedonic scale for sensory analysis.

**Grade**	**Score**
Like extremely	7
Like very much	6
Like	5
Dislike slightly	4
Dislike moderately	3
Dislike very much	2
Dislike extremely	1

**Table 6:** Effect of DOM on physical quality of the collected different degrees of milled rice of BRRI dhan78 variety.

**Parameters**	**Brown rice (0% DOM)**	**Partial milled rice (5% DOM)**	**Full milled rice (10% DOM)**	**Correlation coefficient**
L/B	2.3 ± 0.2^a^	2.5 ± 0.2^b^	2.8 ± 0.1^c^	0.942^**^
*L*∗	61.8 ± 0.3^a^	69.6 ± 0.2^b^	72.7 ± 0.4^c^	0.968^**^
*a*∗	4.5 ± 0.2^a^	1.9 ± 0.3^b^	0.3 ± 0.08^c^	−0.987⁣^∗∗^
*b*∗	23.4 ± 0.4^a^	19.1 ± 0.2^b^	14.0 ± 0.9^c^	−0.992⁣^∗∗^
*c*∗	23.8 ± 0.4^a^	19.2 ± 0.3^b^	13.6 ± 0.7^c^	−0.994⁣^∗∗^
*H*	79.1 ± 0.3^a^	83.8 ± 0.4^b^	88.9 ± 0.6^c^	0.996⁣^∗∗^
Δ*E*	—	9.3	15.0	
*W*	55.0	64.1	69.3	
Hardness (N/m^2^)^†^	3.3 × 10^4^	3.1 × 10^4^	2.2 × 10^4^	−0.866⁣^∗∗^
Chewiness (N/m^2^)^†^	8.1 × 10^2^	8.3 × 10^2^	2.7 × 10^3^	−0.849⁣^∗∗^
Springiness^†^	0.2	0.2	0.5	0.750⁣^∗^
Cohesiveness^†^	0.2	0.2	0.3	0.433

*Note:* Values are the mean ± standard deviation of three replicates. Different alphabets presented on each row indicate significant differences at *p* < 0.05.

^†^Textural attributes of cooked rice.

^*^Correlation is significant at the 0.05 level.

^**^Correlation is significant at the 0.01 level.

**Table 7:** Effect of DOM on proximate composition of the different degrees of milled rice of BRRI dhan78 variety.

**Parameters**	**Moisture (%)**	**Protein (%)**	**Fat (%)**	**Crude fiber (%)**	**Ash (%)**	**CHO (%)**
Brown rice (0% DOM)	10.3 ± 0.2^a^	9.4 ± 0.1*a*	1.3 ± 0.04^a^	1.4 ± 0.2^a^	1.3 ± 0.2^a^	76.3 ± 0.1^a^
Partial milled rice (5%)	10.9 ± 0.9^b^	8.8 ± 0.2^b^	0.7 ± 0.05^b^	0.5 ± 0.1^b^	1.1 ± 0.02^b^	78.0 ± 0.2^b^
Full milled rice (10%)	11.1 ± 0.3^b^	8.0 ± 0.1^c^	0.2 ± 0.1^c^	0.3 ± 0.01^c^	0.7 ± 0.04^c^	79.7 ± 0.5^c^

*Note:* Values are mean ± standard deviation of three observations. Different alphabets presented on each column indicates significant differences at *p* < 0.05.

**Table 8:** Effect of DOM on GI of different degrees of milled rice of BRRI dhan78 variety.

	**Brown rice (0% DOM)**	**Partial milled rice (5% DOM)**	**Full milled rice (10% DOM)**
Glycemic area (mmol/L, min)	148.5 ± 0.001^a^	184.5 ± 0.002^b^	207.0 ± 0.01^c^
Glycemic index (% of pure glucose)	54.4^a^	67.6^b^	75.9^c^

*Note:* Values of glycemic areas are the mean ± standard deviation of three replicates. Different alphabets presented on each row indicate significant differences at *p* < 0.05.

**Table 9:** Sensory quality of different degrees of milled cooked rice.

**Attributes**	**Brown rice (0% DOM)**	**Partial milled rice (5% DOM)**	**Full milled rice (10% DOM)**
Appearance	6.4	7.5	7.2
Taste	5.4	6.6	6.9
Texture	6.4	6.9	7.2
Chewiness	5.5	7.1	7.1
Overall acceptability	6.5	7.5	7.3

## Data Availability

The data that support the findings of this study are available from the corresponding author upon reasonable request.

## Supporting Information

Supporting Information 1 Figure S1. Calibration curve of Vitamin B1.

Supporting Information 2 Figure S2. Calibration curve of Fe.

Supporting Information 3 Figure S3. Calibration curve of Zn.

Supporting Information 4 Table S1. Anthropometric data.

Supporting Information 5 Table S2. Glycemic load.

Supporting Information 6 Table S3. Raw data for glycemic index.
