# Supplementary figures and images for: Effect of Degree of Milling (DOM) on Physicochemical and Nutritional Quality of Selected Rice Variety (BRRI dhan78)
Source: Int J Food Sci. 2025 Jun 27;2025:6034633. doi: 10.1155/ijfo/6034633 (PMC12228569; doi:10.1155/ijfo/6034633)

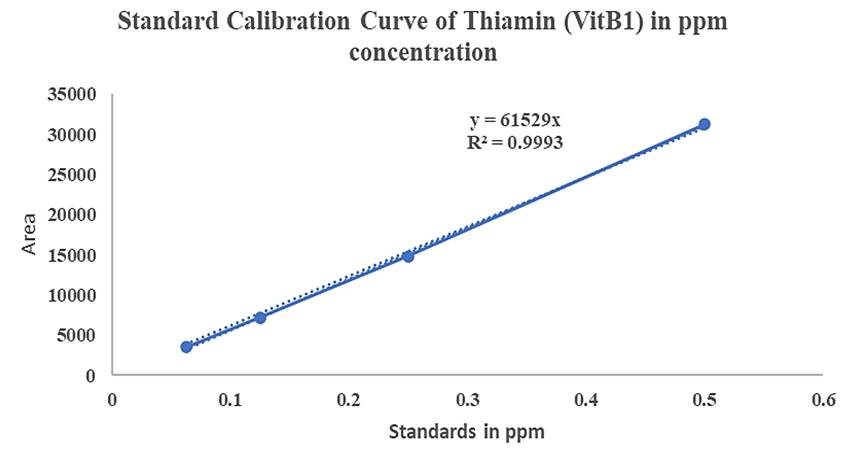

Supplement: Supporting Information 1 — Figure S1. Calibration curve of Vitamin B1. [file 6034633.f1.jpg]

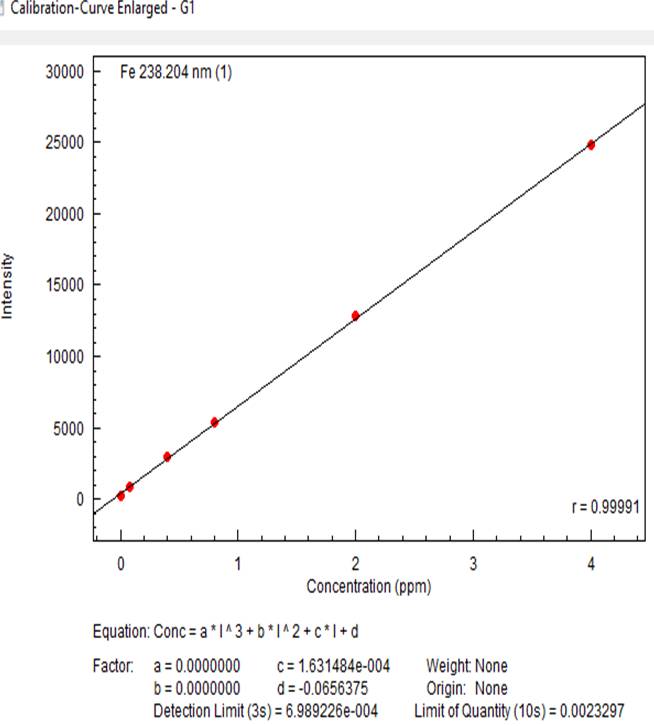

Supplement: Supporting Information 2 — Figure S2. Calibration curve of Fe. [file 6034633.f2.jpg]

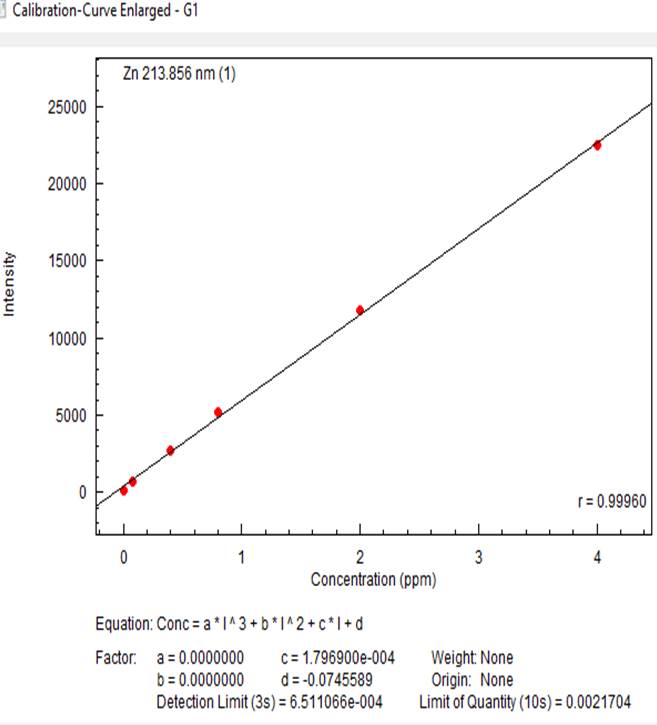

Supplement: Supporting Information 3 — Figure S3. Calibration curve of Zn. [file 6034633.f3.jpg]
