# Supplementary material for: Effect of Degree of Milling (DOM) on Physicochemical and Nutritional Quality of Selected Rice Variety (BRRI dhan78)
Source: Int J Food Sci. 2025 Jun 27;2025:6034633. doi: 10.1155/ijfo/6034633 (PMC12228569; doi:10.1155/ijfo/6034633)
Supplement: Supporting Information 4 — Table S1. Anthropometric data. [file 6034633.f4.docx]

**Supplementary Table 1:** Anthropometric data of respondents

| **Respondent** | **Age (Year)** | **Gender** | **Height (cm)** | **Weight (kg)** | **BMI (kg/m²)** |
| --- | --- | --- | --- | --- | --- |
| 1 | 21 | Female | 160 | 52 | 20.3 |
| 2 | 22 | Female | 165 | 58 | 21.3 |
| 3 | 23 | Female | 158 | 50 | 20.1 |
| 4 | 24 | Female | 162 | 54 | 20.6 |
| 5 | 25 | Female | 167 | 60 | 21.5 |
| 6 | 22 | Male | 155 | 47 | 19.5 |
| 7 | 21 | Male | 168 | 62 | 22.0 |
| 8 | 23 | Male | 163 | 55 | 20.7 |
| 9 | 24 | Male | 157 | 49 | 19.9 |
| 10 | 25 | Male | 170 | 65 | 22.5 |
