# Supplementary material for: Effect of Degree of Milling (DOM) on Physicochemical and Nutritional Quality of Selected Rice Variety (BRRI dhan78)
Source: Int J Food Sci. 2025 Jun 27;2025:6034633. doi: 10.1155/ijfo/6034633 (PMC12228569; doi:10.1155/ijfo/6034633)
Supplement: Supporting Information 5 — Table S2. Glycemic load. [file 6034633.f5.docx]

**Supplementary Table 2:** Glycemic load of rice samples

| **Rice Sample** | **GI**  **(% of pure glucose)** | **Carbohydrate Content**  **(g per 50g rice)** | **Glycemic Load (GL)** |
| --- | --- | --- | --- |
| Brown Rice (0% DOM) | 54.4 | 38 | 20.7 |
| Partial Milled Rice (5% DOM) | 67.6 | 39 | 26.4 |
| Full Milled Rice (10% DOM) | 75.9 | 40 | 30.4 |
