# Supplementary material for: Effect of Degree of Milling (DOM) on Physicochemical and Nutritional Quality of Selected Rice Variety (BRRI dhan78)
Source: Int J Food Sci. 2025 Jun 27;2025:6034633. doi: 10.1155/ijfo/6034633 (PMC12228569; doi:10.1155/ijfo/6034633)
Supplement: Supporting Information 6 — Table S3. Raw data for glycemic index. [file 6034633.f6.docx]

**Supplementary Table 3:** Raw data of Glucose response

| **Time** | **Reference food**  **(pure glucose)** | **Brown rice**  **(0% DOM)** | **Partial milled rice**  **(5% DOM)** | **Full milled rice**  **(10% DOM)** |
| --- | --- | --- | --- | --- |
| 0 | 5.37 ± 0.06 | 5.17 ± 0.06 | 5.20 ± 0.00 | 5.17 ± 0.06 |
| 30 | 11.13 ± 0.12 | 7.33 ± 0.12 | 8.37 ± 0.06 | 9.10 ± 0.10 |
| 60 | 8.73 ± 0.06 | 6.83 ± 0.15 | 7.65 ± 0.14 | 7.83 ± 0.06 |
| 90 | 6.83 ± 0.06 | 6.17 ± 0.15 | 6.67 ± 0.12 | 7.10 ± 0.10 |
| 120 | 6.47 ± 0.06 | 5.07 ± 0.06 | 5.77 ± 0.06 | 6.43 ± 0.06 |
